# Publishing the MISEV guidelines; The editorial process

**DOI:** 10.1002/jev2.12415

**Published:** 2024-02-15

**Authors:** Jan Lötvall

**Affiliations:** ^1^ the Sahlgrenska Academy University of Gothenburg Goteborg Sweden

Extracellular vesicle (EV) research has expanded exponentially over the last 10–15 years, with less than 100 entries in PubMed in 2007 but almost 8000 in 2022, depending on the specifics of the search terms applied. As this meteoric rise began, the field moved at a high pace, with methodology and technology evolving swiftly. This evolution has had notable impact on the stringency of the research produced. When ISEV was established in 2011, it was evident that researchers in the field needed some guidance to improve the quality of published science, as well as advice regarding the technologies being developed to support their work.

The aim of the ISEV leadership has always been to support the positive development of EV research in general. To assist with this, ISEV has published the so‐called MISEV guidelines (Lotvall et al., 2014 and Thery et al., 2018). In the first version, MISEV2014, the word “requirement” was used in the title of the document, which resulted in some criticism, and therefore in 2018 the term “information” was used to better represent the overall goal of MISEV. MISEV guidelines aim to represent the consensus of the field regarding the current state of the art, and they are NOT a set of requirements for EV researchers. MISEV2014 was a short and succinct advisory text on how EV research can provide relatively conclusive results, as well as identifying some caveats that can be avoided in this field of research. Four years later, in 2018, a vast technology development had occurred, with new methods and analytical tools having been introduced to the field. The MISEV guidelines grew significantly, and many authors were recruited to contribute to the document. This effort evolved to the massive MISEV 2018 publication, which is extensively referenced, sometimes as a handbook of EV research. Indeed, if the advice in this document is applied by researchers, the likelihood that the results observed in any study depend on the EVs, are relatively likely.

Since MISEV2018, there have been even further developments in the field, including new methods of EV isolation and technologies to characterize EVs at the single‐EV level. ISEV initiated a process to develop a new and updated MISEV several years ago, and an enormous effort has been employed to develop texts and advice for the version being published in the current issue of Journal of Extracellular Vesicles. The document has 1051 authors, and the whole process of compiling the document has been immense and very challenging but ultimately rewarding.

The editorial approach taken towards the MISEV document, much like the process of compiling the manuscript itself, has been extensive. In the spring of 2023, a full draft of the MISEV 2023 guidelines had been compiled, and discussions between the ISEV board and the Editorial leadership of JEV on how, and when, to submit the document for consideration by the journal ensued. The journal clarified that, although a formal review process is not required for publication of society policy documents, a process to quality assure the manuscript prior to acceptance would be employed. This included several steps.

First, the full‐time scientific editor of ISEV and JEV, Sarah Williams, helped the society to harmonize the texts and the different sections of the manuscript. This required some extensive re‐writing of sections, without losing the message of the text. Further, I, the Editor‐in‐Chief of JEV, contributed at this stage to progress some sections of the text, including certain chapters, but also the abstract. After this, the draft text went back to the ISEV board, which further processed it, before submitting an original version to JEV on 20th June, 2023. As had been clarified during early discussions, the EiC took a leading position in managing the editorial process, which included an extensive quality control procedure that saw over 30 individuals appraise either the entire document, or specified sections within it (Table S1). Many of these individuals, as well as the editorial team, are co‐authors of the document, but this did not refrain any of them from commenting on shortcomings within the submitted version, as is evident in the published feedback in the supplement table of this editorial. On 21st July 2023, the EiC requested extensive revisions to the document based on the comments received during the appraisal process. Friday 3rd November 2023, the society resubmitted a considerably amended version of the work, and a second round of appraisals were performed, this time with 10 individuals who returned their feedback within 1 week. On the 10^th^ of November 2023, the journal requested further minor changes to the document based on some of the quality control suggestions provided by these individuals (Table S2). Finally, on the 28^th^ of November 2023, a further improved manuscript was submitted to the journal and, following consultation with the Deputy Editors of the journal, I, the EiC, approved the manuscript for publication. I am immensely proud of the community, the journal editorial team and those individuals who appraised the document, that this document has finally been published in JEV. I am confident that it will support the wider community with advice and suggestions to develop this field of research further.

MISEV2023 has benefited from the input of a vast number of co‐authors as well as additional appraisal from a large “quality control team” provided by the journal. The previous MISEV guidelines did not benefit from this extended editorial process, but I feel that this has led to the publication of an impressive field consensus document that will be exceptionally important to the community for many years to come. Performing the roles of both Editor and appraiser, as well as being co‐author of any manuscript may be unusual, but I suggest that this process has provided the best possible approach to quality assure the MISEV 2023 guidelines, as well as recognizing the contributions of all those involved. It is my belief that the whole community should be extraordinarily proud of MISEV2023.

## AUTHOR CONTRIBUTIONS

**Jan Lötvall**: Writing—original draft.

## Supporting information

Supplementary Information

Supplementary Information

## References

[jev212415-bib-0001] Lotvall, J. , Hill, A. F. , Hochberg, F. , Buzas, E. I. , Di Vizio, D. , Gardiner, C. , Gho, Y. S. , Kurochkin, I. V. , Mathivanan, S. , Quesenberry, P. , Sahoo, S. , Tahara, H. , Wauben, M. H. , Witwer, K. W. , & Thery, C. (2014). Minimal experimental requirements for definition of extracellular vesicles and their functions: A position statement from the International Society for Extracellular Vesicles. Journal of Extracellular Vesicles, 3, 26913. 25536934 10.3402/jev.v3.26913 PMC4275645

[jev212415-bib-0002] Théry, C. , Witwer, K. W. , Aikawa, E. , Alcaraz, M. J. , Anderson, J. D. , Andriantsitohaina, R. , Antoniou, A. , Arab, T. , Archer, F. , Atkin‐Smith, G. K. , Ayre, D. C. , Bach, J. M. , Bachurski, D. , Baharvand, H. , Balaj, L. , Baldacchino, S. , Bauer, N. N. , Baxter, A. A. , Bebawy, M. , … Zuba‐Surma, E. K. (2018). Minimal information for studies of extracellular vesicles 2018 (MISEV2018): A position statement of the International Society for Extracellular Vesicles and update of the MISEV2014 guidelines. Journal of Extracellular Vesicles, 7(1), 1535750. 10.1080/20013078.2018.1535750 30637094 PMC6322352

