# Supplementary material for: Publishing the MISEV guidelines; The editorial process
Source: J Extracell Vesicles. 2024 Feb 15;13(2):e12415. doi: 10.1002/jev2.12415 (PMC10867691; doi:10.1002/jev2.12415)
Supplement: Supplementary file 1 — Supplementary Information [file JEV2-13-e12415-s002.docx]

| **Full anonymised initial comments for MISEV2023** | |
| --- | --- |
| **1** | Impressions/suggestions:   - In general, the MISEV2023 is complete and read more or less fluency, although some more figures through the text will help to follow the sections. - Maybe the tables with the recommendations could be in lined squared (more visualized) in each section. The recommendations in general are very soft and repetitive in terms of saying “reporting” and not saying what parameters should be reported for the specific sections what will be more useful. - The Preanalytical aspects maybe need more extension and to highlight the importance of having the control of the process of collection of samples and all information from biobanks. - Another aspect not well-covered is the microfluidic development around the EVs. - In general, the figures and tables are useful, maybe Figure 2 could be split in several or in different pages in order to make it bigger at least the part informative about recovery/specificity, and if possible, range of timing will be also very informative. - Line 150. Section …Not or not is… I will reverse and put what it is not first… and what it is after… it will emphasize the what is not is… this is important for the ISEV ¡¡ - Line 218. “***Extracellular particle (EP)*** *is the preferred overarching term for cell-derived multimolecular assemblies in the nanometer to micron size range, including both vesicular and non-vesicular entities: respectively,* ***extracellular vesicles (EVs)*** *and* ***non-vesicular extracellular particles (NVEPs)****.”..* is not clear what the recommendation is ?... and I think it is an important aspect because the concept is introduced or highlighted in this MISEV2023. Maybe require more clarity and advise that although for the vesicle community could sound strong to change to Extracellular Particles it would be more rigorous. Furthermore, normally what we have are mixed populations EPs, EVs, NEVPs in our preparations, so recommendations from the perspective of “Preparations” will be more useful. I think this point could generate a lot of confusion ¡¡¡. In fact, the writing of the whole MISEV 2023 could be compromise and need some cautious writing/ rewriting in order to be coherent with the ISEV recommendations. - Line 286. Section “Collection and pre-processing: pre-analytical variables through to storage”. It looks like that this section is made by contributions of the Task forces of R&S subcommittee, if so, it will be needed to be clearer in the text. … In addition instead of “Tissues”… better “EPs from interstitial space of Tissues”.. we cannot forget the perspective of our Extracellular focus. - A reference to the MOOCIII initiative that is more focused to methodologies could be useful in the MISEV2023. Maybe in section 6. - Abstract. It sounds a little negative and it likes that the field is a little still lost. |
| **2** | Remarks;  This is very comprehensive coverage of these important topics, and overall the recommendations around caution and greater thought on limitations and caveats are very welcome. I particularly appreciate that the broad and hence open to future exciting developments in terms of in vivo work is clearly made; and hence the recommendations reflect generalisms and are fair. There is a lot of emphasis on zebrafish model systems, however, and hence we have a reproducibility problem potentially here because these are highly specialised fish/imaging units. Having seen some of these data, truly verifying the tracking and distribution of EVs as opposed to fluorescence is a significant issue in this domain; and my only suggestion would be to undertake extensive molecular analysis in a spatially resolved fashion of the EV-landing sites in vivo to demonstrate multiple EV-molecules have newly arrived in coincidence with the fluorescence signal. I’m half-suggesting this as a potential additional recommendation around line 1051 , but our zebrafish experts will dislike it as it will be very hard to achieve.  General: Page numbers in the content table need updating. Inconsistent spelling (English/American) for signalling.  Section8-  Lines812,13,14;Lines 629 – 638? text refers to block of release. Should state “inhibition” as block infers a total abrogation of release, which no method yet achieves. Also, the text discusses drugs/genetic manipulations that block and stimulate release; yet the citations given are all focussed on inhibition. We need one/two refs showing enhancement of EV release by manipulations {perhaps this; doi: [10.14814/phy2.14592](https://doi.org/10.14814%2Fphy2.14592)}. Similarly line 821, text is only referring to inhibition of EVs- to showcase roles in function, but for consistency and completeness we should also be open to manipulations around EV-subset stimulation and not just attenuation.  Line330 Recommendations; this says look at other cell biology processes impacted by the manipulation of EV to determine the specificity of the manipulation; Whilst of course this is sensible, the recommendation is far too broad here to the point of being meaningless. I suggest we narrow this- to vital things including confirm cells remain viable / comparably proliferative , and some model secreted factors are explored as unaltered. I also think wrt blocking that add-back of wild type EV into the system to restore functionality can be very useful.  Line258 recommend1; report imaging/labelling method, “and be transparent about the limitations involved”.  Line397. Going forward, inhibition of specific EV ligand-receptor interactions may be required to show that they are responsible for discrete adhesion, signaling, endocytic-initiation, escape and downstream phenotypic effects, for example, the addition of blocking antibodies….  Line903 I felt the 2x recommendations here were rather weak and didn’t refer substantially to the narrative text above. I suggest something akin to   - Carefully consider the suitability of the labelling/reporting system, in terms of its impact on normal operation of cell processes, stability of the EV-association, and longevity of this within an intracellular environment. - Report EV to recipient cell ratios and the physiological relevance of the delivered dose. - Explicit reporting on incubation conditions, exposure time, cell densities and configuration eg 2D/3D - Consider the different steps of interaction with target cells: binding, uptake, content transfer, aiming towards identifying critical mechanistic elements driving the cellular response.   Section9-  Line915; wrt.. “Thirdly, further controls should be employed to rigorously determine whether a specific function is attributed to EVs versus soluble or non-EV co-isolating macromolecular components”. Some mention here perhaps of the difficulties in determining if a factor is EV-associated, or if it is a contaminant. I have not looked earlier in the report whether or not there is ta mention of loosely tethered coronal elements as a challenge to evaluate function and mechanism of function,.  Section10-  Line943; relating to other types of worms where EV have been generated.. 10.1080/20013078.2019.1578116  Line978; care needed, suggesting visualising fluorescent vesicles as puncta can’t resolve single vesicles (yes/true), but the puncta may equally represent a single EV and we must not assume these are EV clusters. We can see single EV (as puncta), but not resolve them.  Line1051; the first recommend item is surely beyond obvious and not needed.  Line1057; include “formulation” in the parameters essential to detail wrt in vivo injection of EV preps. |
| **3** | I believe it is a very nice and well detailed update on nomenclature. Several years have passed since we have started to discuss of vesicles and the complexitiy of the field and of the structures found during these years imposes a further effort so that the expanding community can interact more easily and understand each other. So I agree with you that it is time to introduce new terms to put some order in the vesicle arena.  Here some comments/thoughts  "EV mimetics can be defined as EV-like particles that are produced through direct artificial manipulation of cells. Artificial cell-derived vesicles (ACDVs) is a proposed term for vesicles produced in the laboratory under conditions of induced cell disruption, such as extrusion"  *It is not clear to me if the two terms, EV mimetic and Artificial cell-derived vesicles indicate the same structures or if the intent is to substitute EV mimetics with ACDVs?*  " NVEPs and EVs may have overlapping physicochemical properties, and NVEPs may greatly outnumber EVs in biological matrices. As a result, most EP separation methods result in NVEP/EV co-isolation. Therefore, when EVs and NVEPs cannot be fully distinguished from each other, the term “EP” is recommended"  Do you think that it is possible to suggest in the paper how to distinguish NVEP from EVs so that people may continue to use the EVs term. Perhaps, adding a step of treatment with a detergent may, in a set of experiments, easily discriminate between the two structures?  In the "Recommendation" probably specify when to use Artificial cell-derived vesicles  Finally, what about nomenclature for Plant Evs ? At least we can start here to introduce some term? |
| **4** | As requested, I reviewed sections 8, 9 and 10 of the new MISEV document and I find it excellent, even though it feels slightly generic.  I made some suggestions and comment in the text in the correcting mode and by making notes on margins. I thought this is easier to follow and accept/reject than if I were to extract these comments onto a separate page.  I have also made one suggestion outside of the assigned chapters, namely in the abstract. There I would acknowledge the EPs right at the beginning, as this is increasingly a part of people’s research portfolio, biology and a part of the MISEV document. |
| **5** | MISEV review – sections 8, 9, 10.  Section 8 - EV uptake/release - is it worth reiterating the additional complexity of EV uptake happening at same time as release, so most methods are only measuring net levels at any given moment, which may also vary based on cell culture parameters, and vary for different heterogeneous EV subtypes  For EV release, may also worth mentioning something about specificity and sensitivity of the techniques being developed. As these improve it will help us in understanding the biology (for example by allowing a large number of experiments on smaller volumes of conditioned media).  Section 9 – Functionality  I think it would be good to have a little info here on recommendations around ways of dosing. For example, on the pros and cons of dosing based on ‘EV number’ (which realistically tends to be a ‘particle count’, measured for example by NTA) vs by total protein? Or in cases where engineered EVs are purified, should dosing be defined on total EV number or on the quantity of the functional cargo? There will be no clear answers, of course, but a little discussion on this would be beneficial for the community.  Section 10 – in vivo models.  Looks good. |
| **6** | Overall, sections 4 and 5 of MISEV are very well written. Some minor points:  Lines 343 – recommend reporting all CPPs. There was a lot described in the section above – it would be good to have tables to help the reader easily access and focus on the key points.  I find the third recommendation in section 4.1 a bit out of place (lines 347-349). The effect of CPPs and how they influence EV profile is a separate science in and of itself; most folks reading this won’t be studying this, so it’s irrelevant for most readers. That said, I recognise it does say ‘if possible and relevant’. Perhaps it can left as is, but it just feels a bit odd and out of place to me.  Line 643-644. EVs have been collected from frozen as well as tissues. Given the issues described earlier in the article, that freezing and thawing cells can lead to the artefactual generation of vesicular structures, is it worth adding a comment/caution that further work is needed to understand how relevant these are?  The list of factors (lines 272-275) should be separate by commas, not semi-colons.  Line 429-430 –statement about blood residuals affecting omics analysis – needs a supporting reference  Personally I don’t think it’s absolutely necessary to go into huge detail on the purification techniques, as there are other reviews out there to cover it.  On a separate point – it seems a shame not to use the ISEV-backed ‘history of EVs’ reference in this article (PMID: 34919343), perhaps somewhere in the second paragraph (lines 106-115). |
| **7** | Chapter 4   - Paragraph 1 – very confusing . The terms ‘considerations’ and ‘factors’ are not clear. The paragraph simply relates to parameters affecting quality and quantity during isolation. - Remove ‘ at any time point’ after ‘storage’ - Paragraph 2 – why the need for the preamble about the task force. This is irrelevant to the crux of the guidelines. Perhaps this should be cited as a footnote rather - Overall recommendations in the chapters should be presented as a tables - Section headings – need more elaboration than simple ‘ blood, urine etc’ - Reference to task forces does not belong in body of text – assign aa footnotes rather - The word strongly recommended is featured – are these guidelines no more than recommendations. If this is the case, this could be problematic in publishing in JEV. Careful Rewording is thus recommended.   Specifics comment below for 4.1 and 4.2- other sections ok  Section 4.1   - Paragraph 2 – ‘ duration of culture’ rather than ‘ duration of conditioning’ - Define NVEP - No need for ‘eg short tandem repeat’ - Last sentence – is it strongly recommended’ or a ‘requirement to provide this information’?   Section 4.2   - No need to underline the sentence in para 1 - What is being done to address the separation bias mentioned? This should be stated - Para 2 – what are the simpler, gentler techniques being used? - Para 3- first sentence- grammatically incorrect and needs completion   Chapter 5 – as above for recommendations and task forces. No other comment |
| **8** | The MISEV guidelines will be invaluable for individuals new to scientific investigations, providing a set of recommendations that encompass good common sense rules applicable not only to extracellular vesicle (EV) investigations but also to general scientific research practices. The guidelines emphasize the importance of widely accepted standards in scientific reporting i.e. describing experimental procedures in a manner that allows other labs skilled in the art to reproduce the findings accurately. Many of the recommendations for each technique are aligned with the manufacturer's suggestions and serve as important considerations for users. Consolidating all these recommendations into a single document will be particularly helpful for newcomers to the field seeking to employ some of techniques for their EV investigations.  While the MISEV guidelines encompass general recommendations, they also include some EV-specific considerations, such as the potential risks associated with the use of lipophilic dyes for EV labeling. However, it is unfortunate that the manuscript continues to cite studies that relied on the use of such dyes. I would recommend excluding the citation of Peinado et al. 2012, as this paper has been frequently referenced to support the notion that EVs can home to pre-metastatic niches and promote metastasis. However, the study's conclusions based on PKH labeling of EVs are, at best, premature. Regrettably, this unsubstantiated conclusion has raised safety concerns. Similarly, the second paper by Sheldon et al. utilized DiI, another lipophilic dye prone to labeling artifacts. It is important to note that the first paper did not employ labeled EVs but rather labeled MSCs, which are the source of the EVs. The citation in line 882, 'Studies of this kind have therefore been informative in suggesting potential physiological and pathological roles for EVs in animals, e.g., in suppression of senescence and regeneration (Dorronsoro et al. 2021; Del Campo et al. 2022), angiogenesis (Sheldon et al. 2010) and metastasis (Peinado et al. 2012),' should be revised accordingly.  I suggest that the authors review the cited references and exclude those where the methodology is problematic. Furthermore, it is essential to acknowledge the first report on the referenced issue and attribute credit appropriately, rather than including later "me too" reports. |
| **9** | These guidelines represent a significant step towards standardizing our field of EV research with transparency and rigor. I believe that fostering awareness, implementation and evolution of these guidelines will contribute to our ongoing progress and success of EV research. I have some feedbacks and suggestions which may further enhance the guidelines.  Regarding the definition of EVs, it might be important to clarify whether platelets are considered EVs? It would be beneficial to include one paragraph describing examples of EVs (non-exhaustive), such as apoptotic bodies, vesicles inside vesicles, as well as the shape of vesicles (EVs are not limited to being spherical, sometimes they can have rod shape, tadpole shape, or other irregular shapes).  In relation to the non-protein labeling of EVs (section 7.3), nucleic acid labeling is worth mentioning. The use of membrane-permeant nucleic acid stains has been reported in EV studies (doi:10.3390/ijms18112488; <https://doi.org/10.1098/rstb.2013.0502>; <https://doi.org/10.1167/iovs.61.2.30>). While this approach is being employed in many labs, concerns have been raised regarding vesicle/dye aggregation or non-specificity. Techniques such as over-dilution of samples until the concentration of unbound dye to prevent background interference (<https://doi.org/10.3390/ijms221910510>) would be recommended.  In the recommendations for blood EV processing (section 4.3), I suggest avoiding the practice of keeping blood samples on ice as well and propose that room temperature provides the optimal conditions for preserving their properties. In addition, the bias towards collecting blood from fasting donors to reduce the lipoprotein concentration should be addressed. Other factors such as glucose concentration in the blood and the difference in EV profiles between non-fasting and fasting donors would impact EV characterization. Considering the various approaches available for depleting lipoproteins from EVs, it is recommended to perform rigorous purification as the optimal option.  Finally, there are a few minor editing issues that need attention, e.g. superscript “-1” in the unit mentioned in section 6.1 line 915 and section 6.3 line 997. The first comma should be replaced with a period mark in section 7.3 line 200. An extra word ‘input’ in section 7.2 line 132 should be removed. |
| **10** | 1. Page 1. The abstract is a very ‘public-facing’ part of MISEV. Do we feel it is necessary to single-out ‘commercial separation kits’ for criticism? I mean, the vast majority of methods we use (both commercial and non-commercial) do not yield pure EV as such. Would it be better to use a more generic sentence here such as ‘Although these efforts have had measurable impacts, the EV field continues to be challenged by, among others, techniques that do not necessarily yield pure EVs,…’  2. Page 2. The text in MISEV IS – point 9 ‘.., the basic principles are applicable to all EV sources.’ This I feel assumes we know all the sources of EV. But perhaps we don’t and we should be forward thinking to allow for new things to be discovered. Hence perhaps ‘..the basic principles are most likely to be applicable to all EV sources.’  3. Page 6, Recommendations section, first bullet point. I’m not a fan of using the term ‘ALL CCPs’, as its both confusing (is ALL an acronym used before CCP?) or alternatively a bit ‘shouty’, which is I think not really what MISEV should be about. Perhaps use lower case and underline to emphasise, i.e ‘all CCPs.’  4. Page 8, second paragraph. Is the term ‘immunoglobins’ still in common usage? Perhaps ‘Immunoglobulins’ might be a better fit.  5. Though it is not a significant problem for me, I think the last paragraph could need some attention in its style. For example ‘The resulting document is the best the organizing committee could achieve.’ is a sentence that somehow down-plays the immense effort of those involved in MISEV. Perhaps a better sense would be from ‘The resulting document is presented to the scientific community by the organizing committee.’ The next sentence then gives qualifications and info that judgement calls had to be made.  6. Page 41, line 107, looks like a rogue ‘r’ has crept in at the end of the sentence.  Finally, can I personally thanks all those involved at task-force and manuscript collation and writing levels for, once more, a truly impressive document that distils a huge range of knowledge into a precise and easy-to-follow paper. Well done! Thank you kindly for the opportunity to review and suggest some (minor) alterations, which of course are at the discretion of EiC and authors as to their implementation. |
| **11** | Please find attached file on Table 2 for my recommendation (blue is my recommendation).  Nomenclature text and Figure 1 is OK.  When I overlook the whole manuscript, reference is not appropriate as you know. As many others, I have worried on that this society has not correctly appreciate on original works. |
| **12** | [1] I would suggest to add the following sentences in the section of 4.2 Bacteria and Recommendations (Line 357, Page 5)   - Make sure to check if there is contaminant EV from medium itself. For example, since Luria-Bertani (LB) medium includes yeast extract, there is possibility of existence of EV from the non-bacterial source. - Make sure to check if the isolated EV is not associated with bacterial contamination.   [2] I would suggest to add the following word in the section of 4.3 Blood and Recommendations (Line 427, Page 6)   - Report specific of the blood collection protocol (e.g., blood collection tube, needle gauge, type of anti-coagulant, etc.)   [3] I would suggest to add the following sentence in the section of 5.1 Pre-separation and post-separation storage and Recommendations (Line 667, Page 10)   - Store EV as a highly concentrated form without dilution, if possible.   [4] I would suggest to add the following sentence in the section of 5.2 Separation and concentration (Line 692, Page 11)   - Also, concentration of EV from cell supernatants has been in challenge due to low scalable potential. Recently, tangential flow filtration (TFF) has been employed as a size-based vesicle concentration technique which is able to manage to scalable biological fluids with a reproducible manner (Cells. 2018 Dec 16;7(12):273. doi: 10.3390/cells7120273.). |
| **13** | First of all, thanks for your effort. I really believe that this is an enormous work that the community will really appreciate.  I have tried to give my contribution/help. I think that there is still work to be done but we are close to finishing. The part to work on, more than others, is the “EV separation, concentration and storage” section. In my opinion, MISEV guidelines should go into details of each technique to “drive” people. Said that, I wrote down the structure that it should have, in my opinion, listing the techniques that should be described. However, I do not think that this section should be as long as the “EV source” since there is no task force for each technique. I think that experts in each technique should be invited to write a short paragraph for each of them.  From my side, I looked at sections 4 and 5 as required and also parts of sections 7 (where I felt confident to give suggestions). I added my comments directly to the manuscript. I hope it is ok for you.  I really hope to have been useful and able to give my contribution.  Happy to be part of this community.  Consider 'general recommendations' and don't repeat these in each section.  Section 4 - paragraph 1 It is really hard for me to understand what the authors want to say here.  Section 4 - paragraph 2 '*Of note, for various reasons including how long a given Task Force has been in existence and the activity in that specific research area, some Task Forces are at a more advanced stage than others*' - is this relevant?  Section 4.1 - '*as well as the method (e.g., short tandem repeat) used for confirming the cellular identity*.' I am not totally in agreement with this. In principle yes, but it is insane to ask to do cell characterisation for each new EV paper where cell lines have been used.  Section 4.1 Recommendation 2 : these are guideline, are we really asking to the anyone who publish results about EVs from cells grown using FBS to show what is remaining in the FBS after EV depletion?  Section 4.2 recommendation 3 - *'if possible and relevant'* - In theory this is always a relevant point but is it really feasible to check it?  Section 4.3 - Blood '*it is recommended to measure and transparently report the efficacy of platelet depletion*' since this is a crucial point, i think we should suggest some methods to evaluate this.  Section 4.4 urine - Recommendation 2 - What do you think to list the recommendations here too? At least a summary.  Section 4.5 CSF - 'the current recommendation is to exclude CSF samples that contain >500 erythrocytes/µL from biomarker studies' - what do you think to suggest types of assays to test this?  Section 4 - I have noticed that for the previous sessions mostly about saliva and cerebrospinal fluid, there is an “introduction” part saying what the fluid is and what it does. Here this part is missing. i think we should be consistent.  Section 4.7 - last sentence about the Task Force - is this necessary?  Section 4.9 -consistency issue. we did not say anything about the role of human/animal tissue-derived EVs so I feel that these info about EV from plan do not fit so well here. Or we say more about EVs isolated from human/animal tissue or we remore this part about EVs from plant. Another option is to make a session plant EVs dedicated  Section 5.1 'Pre-storage pre-processing details should be reported, and a reason given if pre-processing cannot be done. Acceptable storage prior to EV separation varies by source.' his has been largely repeated in the previous sections, I do not know if we want to repeat here too.  Section 5.1 - paragraph 2 'cryoprotectants' - should we specify that the presence of cryoprotectants could effect the further experiment results?  Section 5.1 - recommendation 3 - In general I agree with this comments but is it really feasible to test the effect of freezing-thawing in each new study? Isn’t a project itself?  Section 5.2 - kits - I suggest to recommend to show at least a TEM picture to show what the kit is able to isolate (if there are EVs) and with which grades of purity (ideally a picture full of EVs and not background should be showed). This should be recommended also for very broadly used kits  Each isolations method should be briefly mentioned in my opinion saying the pro and cons of each. I do not think that the paragraph about EV isolation methods should be as long as EV source since there are not specific task forces for each of them. - see reviewer 13 word document for proposed structure.  Section 5.2 - last line regarding corona - totlly agree but we should specify, in my opinion that, at the state of the art, it is not so “easy” to investigate the EV corona. Maybe in future it will be something mandatory but not at this moment  Section 7.9 - recommendations - I would suggest to recommend to show EM pictures both close-up and overview and not just a picture of EV showing one or two particles. Authors should give an idea about the composition of EV samples by EM picture putting the reader in the condition to appreciate that the sample is well concentrated. This would be impossible if just one or two EVs are shown.  Section 7.16 - What about to recommend to properly describe the protocol used for protein isolation and quantification? I would suggest to specify that total isolated proteins are loaded on the SDS PAGE. This means that if we are looking for a marker that it is present on 10 EVs out of 10000000000 it will be almost impossible to get positivity. The negative result does not means that the marker, maybe previously identified by another assay (ELISA, mass spectrometry etc) is not present in the EV sample analysed by western blot. |
| **14** | Section 3  Here is some minor points:  Line 240  Although MISEV2018 defined EVs as “naturally produced,” we now remove this term to avoid ambiguities (for example, would “natural” exclude EVs produced in cell culture?). Instead we recommend that the source of EV is clearly defined, for example EVs from cell-culture conditioned media, or EVs from human body fluids including plasma, serum, saliva, urine, tears, bile juice, ascites, etc. EV mimetics can be defined as EV-like particles that are produced・・・・  (rather than stated as “EVs from human plasma”, it is better to define or show example of EVs from body fluids) |
| **15** | Although section 9 about Functional studies of EVs are not enough information, I have no space to make a comment. So I limit minimal comments.  Hope my comments will help for your processing.  Section 8, 9 & 10  A small matter of British English or American  Line 324 analyse to analyze; Line 335 analysed to analyzed; Line 346 normalisation to normalization; Line 350 labelled to labeled; Lines 354, 357 labelling to labeling  Stop here, there are so many and seems set all in British, if so it is fine.  Section 8 8.2 EV release in vivo  Line 369, with some minor point  Recommendations   - Report labelling and imaging technologies.   Consider the effect of EV labelling on the release, not interfere with EVs natural half-life, and properties of the resulting EVs.  Line 915  Recommendations   - Report target cell density, incubation conditions, exposure time - Consider the different steps of interaction with target cells: binding, uptake, content transfer. - Consider labeling EV uptake by high resolution confocal microscope imaging analysis   Section 9 Functional studies  Upon *in vitro* and *in vivo* testing of EV functional potential, including the need for harmonization, establishment of formal potency assays and novel developments for functional testing, JEV paper by Nguyen VVT et al., JEV, 2022 well described, though it is focused on EV therapeutic functions.  Section 10 EV analysis using model organisms  *In vivo* animal models are key to unlocking the potential of EVs biology in toxicology. There are several animal models published after the MISEV2018 to study toxicology, organ transplantation, etc.  ・A toxicity study of exosomes derived from mesenchymal stem cells. Lee J et al., Cytotherapy, 2020. :<https://doi.org/10.1016/j.jcyt.2020.04.044>  ・[Donor extracellular vesicle trafficking via the pleural space represents a novel pathway for allorecognition after lung transplantation.](https://pubmed.ncbi.nlm.nih.gov/35285127/) Habertheuer A, et al., Am J Transplant. 2022 Jul;22(7):1909-1918. doi: 10.1111/ajt.17023. |
| **16** | **I** made some revisions along with some comments. Please see the attached file for details.  Thank you very much for your great efforts!  Abstract – matured should be mature  Section 4.5 – apolipoprotein B-100 should be removed as a marker of blood contamination in CSF.  Section 5 – split the first sentence in two after ‘storage. However…’  Section 5.2 – separate example methods in paragraph 2 with commas.  Section 6 introductory paragraph – change ‘Characterization is challenged by small particle size, heterogeneity of EV size and molecular heterogeneity’ to ‘Characterization is challenged by small particle size, heterogeneity in size and composition’  Section 6, para 4 ‘Orthogonal methods provide measurement of the same parameter derived by’ – delete derived.  Section 6.5 Werwilt et al 2020 - Article information not found in section 13 References.  Section 7.1 A difference in signal intensity might thus mean different particle concentration, epitope density, diameter distribution, or relative abundances of EV subsets. – delete diameter distribution  Section 7.2 – line 1 change utilized for exploited and change considerations for applications.  Section 7.2 line 3 – Zhu et al reference - Please replace this with Tian et al. 2018 (ACS Nano, 2018, 12: 671-80). EVs were not analyzed in the article (Zhu et al. 2014).  Section 7.2 - International Society on Thrombosis & Haemostasis, on not for.  Section 7.2 last para - To avoid confusion, please only keep the MIFlowCyt-EV paper (Welsh et al. 2020). Delete (van der Pol, Welsh, and Nieuwland 2022; Welsh, Tang, et al. 2021; Welsh, Van Der Pol, et al. 2020)  Section 7.2 – evflowcytometry.org is repeated.  Section 7.2 – last recommendation – recommended to the should become recommend to  Section 7.3, last para there is a fullstop where a comma should be after cytometry.  Section 7.4 there is a strange bracket at the end of paragraph 1.  Section 7.6 capitol A Minimal Information About …. Should be lower case.  Section 7.7 Atomic Force Microscopy – should be lower case F and M.  Section 7.8 Total Internal Reflection Microscopy – should all be lower case.  Section 7.8 Willy et al 2021 - The article information in section 13. References is not complete.  Section 7.9 last para – within TEM should be with TEM  Section 7.10 ‘on to a multiplexed array’ should just be ‘on a multiplexed array’  Section 7.15 para 4 replace quantitative PCR (qPCR) with just qPCR. |
| **17** | This is a really well written manuscript and enjoyed the reading.  I made one minor comment on section 6.2. Hope this is helpful.  Section 6.2 In this section, it would be great to include the importance of buffer composition of EV. Viscosity and osmolality of buffer could impact the size of EVs as well as storage stability (<https://onlinelibrary.wiley.com/doi/10.1002/jev2.12238>) |
| **18** | **Comments on new MISEV guidelines document:**  General comment:   - Recommendations for the different fluids in section 4 need to be better balanced. Some are quite detailed, some are quite specific to the fluid, some are very superficial or very general   Section 4:   - Suggestion for alternative title: ‘Collection, pre-processing, and storage’ - Intro, 4.1 and 4.2 need native speaker text editing - Lines 307-311: it should be motivated why we don’t include multi/unicellular parasites, yeast and fungi, which may also be cultured in vivo and are currently studied for EV release/function - Lines 336-340: the reasoning is a bit vague here: ‘we are working on minimal criteria for CCP, but we want you to report ALL CCP’. There are already a lot of criteria in MISEV2018 which should be followed. Then mention this list will be updated in the coming months. - In 4.1 it doesn’t become clear what is new in comparison to MISEV2018. Would be better to not list a lot of ‘open doors’ (like that you need to describe how you cultured your cells), but merely write as an update of MISEV2018 (what have we learned in last 5 years). - 4.2: all other titles of paragraphs concern fluids, so here it would be better to use something like: bacteria conditioned medium - Line 361: why is infected cell culture an ‘in vivo source’? - Line 363-368: this is a statement that there is a risk that separation technology may bias towards specific subpopulations of bacterial EVs: what is the evidence for that? It should also be made more clear what the reader should do with this information: compare different technologies for same sample? - Line 369: ‘densities of EV-rich fractions are not necessarily known’: what is meant here? Densities of fractions can just be measured so that buoyant density of different EV populations is known - Line 370: what is meant with ‘with clear reporting of fractions’? Report the buoyant density values? - Lines 373-374: what is the evidence that the ‘more specific filtration and chromatography methods’ yield more pure EV populations? - Lines 377-378: would be more informative for the reader to mention what kind of methods are currently used - Lines 379-380 ‘if left unfiltered’: why is filtration specifically mentioned here? What if centrifugation has been done to get rid of contaminants: would that be good enough? - Lines 381-382: too much an open door in my opinion - Recommendations: too general in my opinion. These are recommendations for all EV work. What is specific for bacterial EV research? - Lines 429-430: what are recommendations to ‘carefully deplete’ components like immunoglobins and fibrinogen? - Lines 431-442: this paragraph is on EV isolation rather than the pre-analytical variables - Line 463: this is rather a recommendation for EV isolation than for pre-analytics - Line 500: it sounds a bit too simple to just refer to the position paper. This is the MISEV guidelines (= bigger than the position paper)!! - Lines 665-671: for several of the recommendations the word ‘consider’ is used. That’s very vague: what should a non-specialist reader who wants to work on EVs do with that?   Section 5:   - Would indeed be good to add sections on most widely used techniques for separation. If there is not much news to be mentioned with regard to the very standard technologies (UC, density gradient, ultrafiltration, SEC), we may suffice by referring to MISEV2018 and only specify/describe new technologies that gained ground in the last 5 years (eg TFF, acoustic methods etc). |
| **19** | Peer-Review comments for Section 6 and 7  Section 6.1, Row 860: The abbreviation LOD has been used instead of limit of detection before. Therefore LOD should be used here for consistency. Also consider writing NTA, DLS if these abbreviations has been introduced before.  Section 6.2, Row 880: says dynamic light scattering and row 883 says DLS, would recommend to chose to either use the full name or abbreviation and be consistent. The same goes for NTA (row 897 and nanoparticle analysis (row 883 and many more places).  Section 6.2, Row 887. Beside the fact that large EVs might be less abundant, the main problem with the cryo-TEM is the thickness of the ice, which is around 500 nm. That means that a tubular EV over 1 µm in length but below 500 nm in diameter can easily be visualized but a spherical EV (which is the majority) the upper limit is probably 0.5-1µm, Larger EVs will flatten both in TEM and cryo-TEM. Not sure how much in depth you want to go with different microscope techniques for measuring size, but besides negative stain with TEM and cryo-TEM there is also thin sectioning. Here there would be no upper limit restrictions but instead its important to know that when sectioning a EV pellet or a tissue it is impossible to know if you have sectioned a spherical vesicle or a tubal vesicle so caution needs to be taken when claiming sizes from sections. Furthermore even if a spherical EV is measured it is not possible to know if the section has been made at the largest diameter or not. Or if this should be included in section 7.9.  Section 6.2, Although I get what you mean, I think it can be hard for new EV people to understand. I think you can explain a little bit easier/better that techniques such as NTA for example miss a lot of small EVs. This can also be highlighted better in section 6.1. Or in section 7.12.  Section 6.3 Not sure you want to include more references, but there is a paper that have compared measuring EVs with microBCA, BCA, Bradford, Qubit, FluroProfile, NanoOrange and DC Proteins, that might be of value to reference in this paragraph to highlight the differences in for example row 918. PMID: 28577337  Section 6.5. Again not sure if you want to include more references, but there is a paper that shows that different RNA isolation kits isolates different RNA yield and patterns of RNA from EVs. Might be something to highlight here as well, that depending on which kit is used it can result in diffent amount of RNA isolated, besides that the RNA measurement techniques differs. PMID: 22424315. Or if it fits better in section 7.15.  Section 7.1, row 043: Is there an “input” too much here?  Section 7.1 Would it make sense to also recommend reporting the bead to EV ratio and not only total bead number? For example, 10µg EVs per 10^5 beads or 10^5 beads per 0.5ml plasma if EVs has not been isolated prior to analysis. Or is this problematic since we know that 10µg “EVs” can be 2µg EVs and 8µg non-EVs? Should saturation of beads be highlighted as a problem here as well? If you have saturated your beads its hard to detect and increase in signal and the readout might be interpreted as unchanged.  Section 7-2, row 097; Should it be “Recommended to define the upper….” Instead of “Recommended to the define upper…”  Section 7.3, row 106, should not be a full stop after cytometry.  Section 7.6 – row 255 Should this be “Raw data and metadata….” Or perhaps Raw data, data and metadata if both raw data to Pride and similar data repository and data to EVpedia and similar is considered.  Section 7.6 are there also guidelines regarding quantitative proteomics needed (both label and label-free)? Or is this consider general MS guidelines and not EV specific at this point? I think that quantitative proteomics is more common in the EV field then targeted proteomics which is cover a lot in this section.  Section 7.9. See my comment for section 6.2. I don’t think its correct to say that EM “… is capable of detecting the EVs irrespective of size.” Also negative stain will flatten large EVs (beside Cryo-TEM as mentioned before) which will make them look darker and images will become darker and darker, and it will become more and more difficult to ascertain that the object is an EV or any other type of particle.  Section 7.9 Although mentioned in the last sentence of the section it is probably also wise to include in the recommendations that both low and high magnification should be included and that the electrographs should include more than one EV. And that this gives the opportunity to also evaluate the background and possible contaminations.  Section 7.14 row 572 The sentence should start with Resistive pulse sensing (RPS) not Resitive. |
| **20** | I just have an amendment on page 6 as I am unsure very clear for readers.. entering in the field?  maybe examples.. of what we mean by High Throughput methods for quality and quantity?  With all the types of EVs from so many sources that we have now it starts being a tuff job  Thanks to all |
| **21** | Overall this is well written. However, it does appear in parts that it has been written by different groups. Examples of this include reference to different task forces, particularly in section 4. I recommend the discussion of Task Forces, their outputs and aims are moved into a separate section to improve the flow of information.    The manuscript is big, and one wonders how someone new to the field will navigate all of this information. There needs to be a section on ‘how to use MISEV2023’ which could be inserted before section 3 (Nomenclature). There needs to be some thought as to the usability of this large document. A checklist might be useful for this.    The order of items in section 7 needs to be considered more as they are presented in a random order. Perhaps more well used techniques (eg NTA, TRPS, Western blotting) should come first in this section rather than at the end.      Minor comments:   - Line 150 - What does ‘citation-supported introduction to EV research’ mean? - Line 153 – how will reviewers and editors use this very large document? - Line 162 – should add that this is relevant in the appropriate clinical setting (eg EVs prepared within a clinical facility) - Line 196 - Mention is made of the vote by the EV community in 2011 but there is no reference for this – and the vote was for a ‘preferred term’, was it not? - Line 206 – I find this sentence slightly confusing. Is the proposition that EVs are referred to as EPs? - Table 2 is useful - Use the abbreviations after the full spelling of the items in the recommendations - Lines 279 to 294 could be condensed. - Line 331 – it is recommended here to confirm cellular identity using, for example, genetic confirmation. However this is not a recommendation in the summary section. Perhaps instead a reference could be used for best practice in confirming cell line identity. - Line 370 use ‘established’ rather than ‘worked out’ - Line 392 – mention is made of task force when this hasn’t been mentioned earlier on – this sentence could be removed or the Task Force mentioned earlier in the bacterial section. - Line 500 – needs a reference for the ISEV Position Paper mentioned - Line 584 – do you need this text describing the Task force aims? Not included for others… - Line 652 – this section could be condensed – perhaps included in section 4.10 as an example? - Line 684 – what does ‘strict separation’ mean? - Line 1086 – website name is duplicated - Section 7.3 – style of this section is quite different to other sections with numbered steps included – this should be rewritten similar to section 7.4 (third paragraph) - Line 1920 – mention is made of ~5700 members of the ISEV community – were these individuals or pooled from various lists? - Table 2 – needs a definition for Bacterial EVs - Table 4 – needs rating for Ciliated sensory neurons (as is grouped separately to others in this group) |
| **22** | Below, my comments on Section 3 of MISEV.   1. The recommendations on EV nomenclature are consistent with the history and evolution of ISEV recommendations and expert consensus, including as measured by the MISEV2018 survey and last year's survey for MISEV202X. More than 99% of survey respondents agreed (with or without comments) with the initial draft of this section. No major changes are warranted at this stage. 2. An important part of the EV recommendations is how additional "operational" terms are deployed when this is deemed necessary: terms must be clearly defined and (where relevant) associated with accurate measurements. E.g., "Small" and "large" don't mean anything and are unnecessary unless they are clearly defined and supported by data. I hope that this is clear; if not, I am happy to help with re-wording. 3. For the NVEP recommendations, I agree with the underlying reasons for making them and think that this part is overall OK and consistent with the community feedback. At the same time, I have two concerns. I hope these concerns are addressed in the current draft, but if not, I would consider some minor clarifications. a)The first concern is that MISEV not impose a new nomenclature with too much force on researchers of particle types that are technically outside, albeit closely related to, the EV field. ISEV is an EV society, not a lipoprotein or protein aggregate society. We would not propose to define HDL and LDL, etc., since these particles are already well defined, and we should also avoid imposing proposed new types like supermeres and exomeres, which are poorly defined and, some would say, may not be distinct or reproducibly obtained entities. Yes, we can recommend the term "NVEP" as a catch-all, and correctly point out that EVs ARE EPs (so "EVPs" is actually incorrect and should not be used unless as "EVs and other EPs"). I think the section gets this right, but it might be worth moderating. b)The second concern: there is probably no way to purify EVs to homogeneity, or to confirm that absolutely no co-isolates are in a preparation, so if MISEV is interpreted as "always use the term "EP" if NVEPs may be present", no one will ever be able to use the term "EV" again. I hope this is also clear, but, if not, we need to make sure that it's still OK to say "EV" or "EV preparation" and not change the entire future literature on EVs to "EPs". We can't be so strict as to eliminate our field! 4. My final comment is a recommendation for the entire document, not just nomenclature. As in MISEV2018, it would be useful to measure the level of agreement on each major section using the final authorship confirmation survey. This would allow readers to identify where there is broad agreement and where there is a diversity of opinion amongst the co-authors. |
| **23** | **MISEV 2023, review of sections 4 and 5**  Section 4:   - Introductory section, mentioning task forces, Line 280-295: please not only provide a link to the ISEV webpage, but elaborate a bit on the specific task forces in place. My recommendation would be a concise table listing task force name, aim, fundamental recommendations, link to website, etc. - Section 4.1 “Cell culture conditioned media”, Line 307-350: I suggest a standing recommendation to always provide a validated test that cells used for EV isolation in any study are tested mycoplasma (or other microorganism) free – of course only applicable if a study was not about intracellular microorganisms and their EVs. - Section 4.2 “Bacteria”, Line 352-394: Please mention the different sources of bacterial EVs, such as feces or bacterial cultures. - Section 4.5 Line 506. “. As” should be “, as”. - Section 4.5 Line 527, “genotype” may be extended to “genetic and epigenetic profile”. See the reference: DOI: 10.1126/sciadv.abb542 and doi.org/10.1186/s13195-023-01216-7 - Section 4.5 Line 536. In our mouse experiment, only 1-2 µL CSF could be drawn and we had to dilute with PBS prior to downstream separation/analysis. Thus, I would suggest changing to “use of dilution buffer including additives”. - Section 4.6 Line 547. The composition of saliva is a bit overlapping. May be changing to “Saliva consists of water (94-99%) as well as multiple components from the host, ingested food and bacterial such as cells, cellular debris, soluble proteins, electrolytes, and EVs.” - Section 4.7 Line 574. SF EVs from mice, important preclinical model, was studied as well. See the reference: 10.1172/jci.insight.125019 - Section 4.8 Line 605. May mention lipoprotein as the highly abundant confounding protein as well. - Section 4.9 Line 650. Proving the source of EVs (pre-existing or released by cells upon tissue disruption) might be unnecessary as long as they bear biological/diagnostic functions. The more important issue, in my opinion, is to highlight the functions of EVs versus soluble proteins. - Section 4.10 “Other sources”, Line 676-679: Name specific other EV sources/biofluids that are not included here due to the lack of task forces, such as peritoneal lavage, tears, sweat, feces (not mentioned in bacterial EV section either), vaginal lavage, or semen.   Section 5:   - Section 5.1 Line 697-716. Maybe briefly state that storage conditions might affect the biological functions of EVs and provide an example. - Section 5.2 Line 762. The recovery could also be calculated as the amount of EV marker protein (or a facile reporter protein) versus particle counts. See the reference: https://doi.org/10.1080/20013078.2020.1800222 - Section 5.2 “Separation and concentration”, Line 731-789: I suggest either keeping a similar structure like for section 4, based on the EV source, or structuring this chapter by separation methods. Different sources require different separation techniques, however, there are certain overlaps in the use of methods between sources, thus a focus on each method might be more concise. Please also align with the 2018 update focusing on the recovery/specificity of each method. |
| **24** | MISEV 2023 Review of Chapters 8-10  For the most part, Chapters 8-10 are well written and comprehensive. These Chapters cover from the biogenesis and uptake of EVs to functional studies using in vitro and in vivo models. The authors did an excellent job reviewing these topics in detail. A few areas need references particularly the first 2 paragraphs in Section 8.3 EV interaction with cells. Chapter 8 may benefit from additional *in vivo* models to study release, as well as expanding on the studies done in Drosophila. Perhaps comparing the benefits of these different systems would be of interest. Section 8.2 “EV interaction with cells” is not as cohesive. It may benefit the flow of this section to have the paragraphs reorganized (where numbers refer to the paragraph order at present): 1, 4, 2, and 3.  Minor:   1. Line 812: Change “block” to “inhibition” or “blockage” in the sentence, “In MISEV2018, approaches used to analyse the functions of specific EVs involving the block or stimulation of EV secretion…” 2. Line 926: Include “time course” in the sentence, “Use of dose-response and time course studies…” 3. Line 1054: Correct “e.g.,” 4. Lines 1055-56: “…the distinction between EV uptake and function.” These are already very different events and it’s not clear why this phrase is needed. 5. Section 10.2 (lines 1034-1037): The sentence, “Parabiosis in mice...” doesn’t seem to fit here and disrupts the flow. |
| **25** | 1. In the sentence “Use of biogenesis-related terms such as “exosome” and “ectosome/microvesicle,” I suggest deleting the term “microvesicle” because microvesicle is by no means synonymous with “ectosome”; it is just one subtype of ectosomes 2. It should be clarified if EVs produced by cells transfected or transduced to express certain proteins in association of EVs (“engineered EVs”) fall into the category of EV mimetics based on the definition found in the manuscript (“EV mimetics can be defined as EV-like particles that are produced through direct artificial manipulation of cells.”). Maybe it would be worth introducing a separate term “engineered EVs”. 3. “Lipoprotein subtypes overlap in size or density with EVs and some have been reported to associate with RNAs.” I suggest modifying to “have been reported to associate with RNAs or EVs.” |
| **26** | Overall the MISEV guidelines have significantly improved since the previous versions (I already read twice and delivered extensive feedback before). Related to content I thus have no further changes to request.    However I would like to note that:   1. although I am in favor of the highlighted recommendations in each subsection, I believe these still require fine-tuning to ensure they deliver a consistent message at similar levels throughout the manuscript. 2. although readers are mainly referred to MISEV2018 for separation/concentration (I do hope the readers will do so because this section disappears between the extensive nomenclature, pre-analytical and characterization sections), I believe it remains important to stimulate researchers to collect and evaluate EV-enriched but also presumable EV-depleted fractions, which will contribute to understanding the performance of the separation/concentration steps but also contribute to understanding EV composition/function.     Some details to end with:   1. page 7, section 4.3, line 9: Dhondt et al 2020 should be Dhondt et al 2023. 2. typos are to be corrected throughout the manuscript. |
| **27** | The new MISEV2023 guidelines are an updated version of the 2018 guidelines and are meant to harmonize reporting on EV data. This is again an impressive piece of work, though I see it a bit more stringent compared to previous versions. I have some recommendations for improvement, see below. Please note that some sections were just scanned as I am not an expert (or even familiar with) all techniques discussed in these sections  Major comments  1: Overall the sections I reviewed (6 and 7) are based on hypothetical “pure” EV isolations/preparations, although NOBODY knows what this would be. I would recommend to leave room for pragmatism, and be careful not to get too much caught up in disclosures/conditions. I think this would make the paper, and the research field, more inclusive, instead of exclusive.  2: In the recommendations, I suggest to avoid the word “should”… keep it with suggestions. If EV paper would include everything that they “should” report, the Mat and methods sections would become way too lengthy. I suggest that authors state that they are aware of the limitations of their approach, and discuss that if relevant. All necessary details may be reported in a suppl file or in repositories like EV-TRACK.  3: MISEV is supposed to be a recommendation on reporting guidelines, right? Especially in section 7 I see a focus on guidelines how to set up experiments. I feel this could be less restrictive and leave more freedom here, as long as proper controls are taken along and the assays fit the context of use/fit for purpose  Minor comments  Line 799: Section 6, para 2 *If making claims about an EV preparation, the extent to which a sample will need to be characterised to justify the claims may depend upon the source of the material* - depends also on also isolation/enrichment method  line 803: Section 6, para 3 *Overall EV composition* overall composition: in the source or the preparation?  Line 805-806: Section 6, para 4 *Just as no single molecular class measurement can quantify all EVs, there are also no universal molecular markers of EVs or EV subtypes. Markers must be chosen based on source- and type-specific evidence. Currently, no generic marker is known to identify all EVs irrespective of source.* although not absolute measures, they can be used to compare between samples, for example within an experiment  Line 816-7: Section 6 para 5 - *Orthogonal methods provide measurement of the same parameter derived by different means and therefore are unlikely to have the same biases; e.g., optical vs. non-optical derivation of diameter.* please rewrite, sentence is confusing now  Line 818: Section 6 para 5 *Characterization of EV samples using orthogonal methods is critical to provide evidence* “evidence”, should that be “confidence”  Line 822: Section 6 para 5 *A framework for reporting EV data has been previously developed and updated in the form of EV-TRACK (Consortium et al. 2017; Roux et al. 2020)* reference “consortium”, is that correct?  Line 830: Recommendations - number is not mentioned before in this section  Line 832: Section 6, recommendation 2 - replace “and/or” by a comma  Line 836: Section 6, recommendation 4 - add “especially contamination by dead/broken cells or parts thereof”  Line 843: Section 6.1, para 1 - *However, it is often unreliable, since many techniques lack specificity for EVs and sensitivity for all EVs*. “unreliable”, maybe add some nuance, as for relative comparisons all requirements are not absolutely necessary  Line 850-2: Section 6.1, para 1 *The ISEV Rigor and Standardization EV Reference Material Task Force recently outlined the considerations in measurement techniques, along with the challenges faced by the field in moving towards traceable measurements, for the development and reporting of well-characterized EV reference materials,* difficult sentence, please rewrite  Line 853: Section 6.1, para 1 use abbreviation: “LOD”, additionally, I suggest to mention a formal definition of LOD.  Line 868: Section 6.1 para above recommendations - has the abbreviation “EP” been introduced?  Line 874: Section 6.1, recommendation 3 - I understand that the term “EPs” may be considered, however I feel this will not be broadly implemented (like the term exosomes, which is still used to refer to EVs….)  Line 877: Section 6.2, para 1 - “assumptions”: EVERY assay is based on assumptions, like reaction speed, correlation to (pure) standards, etc…  Line 879: Section 6.2 para 1 - ehhh… are EVs not spherical? Or is physics also an assumption?  Line 889: *The ability to quantify low contrast EVs below 100 nm may also be a limiting factor.* Last sentence of this paragraph may be removed  Line 919: Section 6.3, para 1 what are more specific methods vs “less specific methods””  Line 916-926: Section 6.3, para 1 - depends also on source and isolation technique  Line 934: Section 6.4 - something about LOD for lipid quantification could be helpful  Line 946: Section 6.5 – para 1 This paragraph needs a better introduction sentence. Overall, the section on total RNNA is not really constructive and is covered in the later section (7.15). Now only concerns are addressed here…  Line 995: Section 6.7 - Are there no recommendations for section 6.7?  Line 997: If section 6.8 was not there, I would not miss it. Consider deleting.  Line 1011: Not sure if section 6.9 has value for MISEV2023, this is very general and can be omitted  Line 1038: Section 7.1, para 1 - “semi-quantitative only”, is should be realized that this in principle is a “bulk” measurement, displaying avg intensity of a lot of particles  Line 1056: *The detection of single EVs using flow cytometry has been utilized for several decades and resulted in the development of many considerations.* this line is obsolete  Line 1064: Section 7.2, para 1 - is the paper by van de Vlist, Wauben and Nolte-‘t Hoen cited in this section?  Line 1078-9: Section 7.2 para 2 please rephrase, difficult sentence  Line 1087: Section 7.2, para above recommendations - introduce abbreviation “FC”  Line 1089: Section 7.2, para above recommendations check website  Line 1143: Section 7.4 Please explicitly make clear that genetic tagging will label only a subset of EVs  Line 1206: Section 7.6, first sentence - EV-derived, is that the best word. Maybe EV-associated, or EV-resident?  Line 1214: Section 7.6, para 1 - not sure if the general readership will understand that “ions” are the charged peptides resulting from protein digestion, suggestion to include explanation.  Line 1479: Section 7.12 - MISEV2018 guidelines should be referenced I think, those were very clear  Line 1619: Section 7.15, para above recommendations - this paragraph is very general. Overall, there are many general guidelines for DNA/RNA analyses, including repositories for data etc. I feel this entire section can be much more compact  Line 1666: Section 7.16, para 2 - see above, for antibodies in principle a cat nr would suffice. |
| **28** | Section 8.1 recommendation 1 - 'secretory' Shouldn´t be mentioned?  Section 8.2, para 1 The fading of fluorescent probes such as GFP in acidic compartments  Section 8.3 para 1 a paper proposing that the release of EV content into recipient cells can occur through the formation of Connexin-containing " channels (PMID: 26285688)  Section 8.3 para 4 - I would tone down this sentence since lysosomes have been consistently shown to act as signalling hub. We have some evidence that EV can modulate some lysosome-associated signals. |
| **29** | The new MISEV2023 guidelines are an updated version of the 2018 guidelines and are meant to harmonize reporting on EV data. This is again an impressive piece of work, though I see it a bit more stringent compared to previous versions. I have some recommendations for improvement, see below. Please note that some sections were just scanned as I am not an expert (or even familiar with) all techniques discussed in these sections  Major comments  1: Overall the sections I reviewed (6 and 7) are based on hypothetical “pure” EV isolations/preparations, although NOBODY knows what this would be. I would recommend to leave room for pragmatism, and be careful not to get too much caught up in disclosures/conditions. I think this would make the paper, and the research field, more inclusive, instead of exclusive.  2: In the recommendations, I suggest to avoid the word “should”… keep it with suggestions. If EV paper would include everything that they “should” report, the Mat and methods sections would become way too lengthy. I suggest that authors state that they are aware of the limitations of their approach, and discuss that if relevant. All necessary details may be reported in a suppl file or in repositories like EV-TRACK.  3: MISEV is supposed to be a recommendation on reporting guidelines, right? Especially in section 7 I see a focus on guidelines how to set up experiments. I feel this could be less restrictive and leave more freedom here, as long as proper controls are taken along and the assays fit the context of use/fit for purpose  Minor comments  Line 799: Section 6, para 2 *If making claims about an EV preparation, the extent to which a sample will need to be characterised to justify the claims may depend upon the source of the material* - depends also on also isolation/enrichment method  line 803: Section 6, para 3 *Overall EV composition* overall composition: in the source or the preparation?  Line 805-806: Section 6, para 4 *Just as no single molecular class measurement can quantify all EVs, there are also no universal molecular markers of EVs or EV subtypes. Markers must be chosen based on source- and type-specific evidence. Currently, no generic marker is known to identify all EVs irrespective of source.* although not absolute measures, they can be used to compare between samples, for example within an experiment  Line 816-7: Section 6 para 5 - *Orthogonal methods provide measurement of the same parameter derived by different means and therefore are unlikely to have the same biases; e.g., optical vs. non-optical derivation of diameter.* please rewrite, sentence is confusing now  Line 818: Section 6 para 5 *Characterization of EV samples using orthogonal methods is critical to provide evidence* “evidence”, should that be “confidence”  Line 822: Section 6 para 5 *A framework for reporting EV data has been previously developed and updated in the form of EV-TRACK (Consortium et al. 2017; Roux et al. 2020)* reference “consortium”, is that correct?  Line 830: Recommendations - number is not mentioned before in this section  Line 832: Section 6, recommendation 2 - replace “and/or” by a comma  Line 836: Section 6, recommendation 4 - add “especially contamination by dead/broken cells or parts thereof”  Line 843: Section 6.1, para 1 - *However, it is often unreliable, since many techniques lack specificity for EVs and sensitivity for all EVs*. “unreliable”, maybe add some nuance, as for relative comparisons all requirements are not absolutely necessary  Line 850-2: Section 6.1, para 1 *The ISEV Rigor and Standardization EV Reference Material Task Force recently outlined the considerations in measurement techniques, along with the challenges faced by the field in moving towards traceable measurements, for the development and reporting of well-characterized EV reference materials,* difficult sentence, please rewrite  Line 853: Section 6.1, para 1 use abbreviation: “LOD”, additionally, I suggest to mention a formal definition of LOD.  Line 868: Section 6.1 para above recommendations - has the abbreviation “EP” been introduced?  Line 874: Section 6.1, recommendation 3 - I understand that the term “EPs” may be considered, however I feel this will not be broadly implemented (like the term exosomes, which is still used to refer to EVs….)  Line 877: Section 6.2, para 1 - “assumptions”: EVERY assay is based on assumptions, like reaction speed, correlation to (pure) standards, etc…  Line 879: Section 6.2 para 1 - ehhh… are EVs not spherical? Or is physics also an assumption?  Line 889: *The ability to quantify low contrast EVs below 100 nm may also be a limiting factor.* Last sentence of this paragraph may be removed  Line 919: Section 6.3, para 1 what are more specific methods vs “less specific methods””  Line 916-926: Section 6.3, para 1 - depends also on source and isolation technique  Line 934: Section 6.4 - something about LOD for lipid quantification could be helpful  Line 946: Section 6.5 – para 1 This paragraph needs a better introduction sentence. Overall, the section on total RNNA is not really constructive and is covered in the later section (7.15). Now only concerns are addressed here…  Line 995: Section 6.7 - Are there no recommendations for section 6.7?  Line 997: If section 6.8 was not there, I would not miss it. Consider deleting.  Line 1011: Not sure if section 6.9 has value for MISEV2023, this is very general and can be omitted  Line 1038: Section 7.1, para 1 - “semi-quantitative only”, is should be realized that this in principle is a “bulk” measurement, displaying avg intensity of a lot of particles  Line 1056: *The detection of single EVs using flow cytometry has been utilized for several decades and resulted in the development of many considerations.* this line is obsolete  Line 1064: Section 7.2, para 1 - is the paper by van de Vlist, Wauben and Nolte-‘t Hoen cited in this section?  Line 1078-9: Section 7.2 para 2 please rephrase, difficult sentence  Line 1087: Section 7.2, para above recommendations - introduce abbreviation “FC”  Line 1089: Section 7.2, para above recommendations check website  Line 1143: Section 7.4 Please explicitly make clear that genetic tagging will label only a subset of EVs  Line 1206: Section 7.6, first sentence - EV-derived, is that the best word. Maybe EV-associated, or EV-resident?  Line 1214: Section 7.6, para 1 - not sure if the general readership will understand that “ions” are the charged peptides resulting from protein digestion, suggestion to include explanation.  Line 1479: Section 7.12 - MISEV2018 guidelines should be referenced I think, those were very clear  Line 1619: Section 7.15, para above recommendations - this paragraph is very general. Overall, there are many general guidelines for DNA/RNA analyses, including repositories for data etc. I feel this entire section can be much more compact  Line 1666: Section 7.16, para 2 - see above, for antibodies in principle a cat nr would suffice. |
| **30** | First of all, I must say that I am not a super at ease with MISEV 2023, and to be honest, I am also not a super fan of 1000 authors papers in general. To me it ends up in a chaos … being more a way for many to gain citations rather than a collective effort that seriously/critically pushes a field forward. To be frank, I am afraid this 2023 edition won’t really help the field to gain in credibility…it could even favor some form of ‘inanity’.  To stay on a constructive note, considering the expansion of the field, I think it may become pertinent to go for a format like ‘Current protocols in … EV research’ with sections written and regularly edited by groups of respected experts (I mean people with long experience, important records and/or at the frontline for the matter of the section). With that, reviewing and criticism by juniors (in the field) may be considered as I think it would help reaching good quality, since at the end of the day, the goal is insightful up-to-date education for new comers.    Anyway, here my suggestions for Section 8.    8.1. Add the recent review of Weaver and colleagues (Nat Rev Mol Cell Biol 2023) which is an educative, up-to-date and non-dogma biased review (my humble opinion)… add at least syntenin silencing and PLD2 silencing or inhibition as methods to decrease EV production with ad-hoc refs.  When indicating request for complementary methods, add examples (e.g. different siRNAs, chemical inhibitors and/or gain of function/overexpression).  I would not necessarily propose EV number as a standardization method knowing the limitation of current methodologies to fractionate and measure these particles, and also because this type of standardization would mask non-controlled pre-analytical parameters.    Under recommendations:  *‘Consider whether blocking a specific EV production pathway may lead to a change in other EV release mechanisms’.* Considering the mechanisms are not completely understood, vary between cell types and pre-analytical conditions and considering that a single paper is not meant to revisit/validate/invalidate all the findings of others… I would drop this recommendation. This would also avoid opening the door for abusive expectations from some reviewers. Moreover, knowing for ages that membrane exchanges are interconnected … I wonder what would be the added value to show that pathways governing these membrane exchanges are influencing each-others…    8.2  Considering MISEV is not meant to replace common sense in a given domain, I would drop the recommendation *‘Consider the impact on other secretory processes of genetic manipulations used to suppress EV release; data should be interpreted conservatively taking in to account the limited selectivity of many approaches.’*  Maybe suggest if needed, to improve problems related to over-expression by proposing CRISPR edition, add e.g. GFP-tag to the endogenous gene.    8.3  I would drop the following paragraph because too premature. *‘Since MISEV2018, studies have suggested that EV uptake occurs at a low rate (Bonsergent et al. 2021; Somiya and Kuroda 2021a, 2021b), at least in some target cells and a high ratio of EVs to target cells would be required to visualize this process (Jurgielewicz, Yao, and Stice 2020; Ragni et al. 2019). One report focusing on specific cell type and using subcellular fractionation approaches, however, suggested that 30% of EV proteins that were taken up were transferred into the cytosol via a mechanism involving acidification and membrane fusion (Bonsergent et al. 2021). New approaches for assaying cargo delivery (including endosomal escape) have been developed since MISEV2018, e.g., anti-GFP fluobodies (Joshi et al. 2020), proteolytic cargo cleavage (Perrin et al. 2021), split-luciferase reporters (Somiya and Kuroda 2021a), CRISPR-Cas9 reporters (de Jong et al. 2020), Cre-reporters (Borghesan et al. 2019) and trans-activator delivery (Somiya and Kuroda 2021b). By labelling specific EV subtypes, blocking their biogenesis in secreting cells, and using the novel cargo delivery analysis methods, it may be possible to determine how EV-target cell interaction mechanisms vary between different EV subtypes and EV donor-acceptor combinations.’*  If the authors want to propose methods, they should do so and describe the methods used in these papers and stress on their respective limitations (and limited conclusions). Basically the reader would be interested to discover how one can measure the EV-attachment, uptake, and escape in a reliable manner.  I also think the section deserves the endosomal escape process to be addressed: state-of-art, challenges, and what is next. Recommendations to be considered. |
| **31** | **Comments to the Authors:**  **Section 3**   1. In Line 215 and in several other places in the manuscript, it states that EVs cannot replicate on their own. Is there any evidence favoring or disputing this fact? Seems rather odd to even mention it. EVs this way may classify them in a misleading way without any basis for such consideration. Either a citation should be included that supports this notion, or the statement entirely removed. 2. Line 230: It is unclear what the authors mean by ‘terms that highlight specific aspects of biogenesis’. This statement should be revised to clarify if this is in reference to ectosomes, exosomes, and apoptotic bodies. 3. Figure 1: It would be beneficial to label this figure as ‘current hierarchy of EP nomenclature’, as the nomenclature will likely evolve with time and this statement needs to progressive in nature with an open mind. 4. Table 2: The authors state that MISEV2023 is not intended to tell people how they should perform EV research, but coloring a table as green/yellow/red with ‘recommended/recommended with caution/discouraged’ does not align with this intention. I recommend revising this table to relabel the usage section to instead reflect which terms are more general vs. specific. 5. In general, I find the language to have a tone of being “lectured about”. This statements that use ‘likely’, ‘suggest’ and ‘offer guidance’ feels more like a dialogue. This can be easily incorporated in my opinion.   **Section 8/9/10**   1. Section 8 Conflicting suggestions are reported within this section: line 773 suggests that inhibition of EV release is not specific, in line 812 the authors recommend blocking biogenesis pathways to inform on EV-target cell interaction mechanisms. This should be revised to be consistent or include caveats of such approaches. Also, it should be clearly stated that we still not have any method to completely shut down EV biogenesis, small EVs or Exosomes included. This is really confusing to the field, and we need to make sure some select dogmas with non-absolute data are not conveyed as defining. This will be of great help to the community. 2. Section 8 Line 820: Genetic approaches to inhibition of receptor-ligand interactions should be included as blocking antibodies and pharmacological inhibition are in many cases not specific. This is truly important for our trainees and those entering the field of EV research. 3. Section 8 Line 827: The recommendations include target cell density, incubation conditions, and exposure but there is no discussion of how such parameters may influence the observed interactions between EVs and cells. 4. Section 9 Line 834: Media that has not been in contact with cells is likely not a sufficient negative control for many experiments. Some discussion of showing cell type specificity of EV-induced phenotypes should be included here, as was for biofluids derived EVs. 5. Section 9 Line 838: It would be helpful to include some recommendations of how to address the contribution of EVs vs. soluble/non-EV components to recipient cell function. 6. Section 10 Table 4: It would be helpful to include a column in this table for EV detection resolution and model limitations. |
| **32** | This manuscript is a massive undertaking by the community that overall should be supported and published. It is important, and helpful, for EV researchers at all level.  I have a few major concerns:  Firstly, this is a Minimum Requirement (or perhaps minimal) for EV research, but the manuscript describes a vast array of advanced and perhaps unnecessary techniques for EV research. Instead of being a “minimal guideline”, it has become an “advanced guideline”, or an encyclopedia of EV research approaches. This is of course helpful in many ways, but it is questionable whether this is minimal guidelines.  An important portion of the manuscript is dealing with nomenclature, and enters the murky waters of non-vesicular nanoparticles, and attempts to build nomenclature beyond EVs. This does not seem appropriate for an article that otherwise is giving no advice on how to study non-vesicular (naturally released) nanoparticles. I suggest this section is focusing exclusively on EVs, and avoids the trap of particle nomenclature.  Another huge caveat is chapter five, which is not describing or suggesting methods/techniques to isolation / purify vesicles. Not even ultracentrifugation is described. This must be changed for the MISEV2023 guidelines to get the impact it deserves.  The manuscript is unfortunately littered by grammatical errors, which of course can be rectified by professional editing. |

Supplementary Table 1. Full anonymised initial comments for MISEV2023.
