# Supplementary material for: Publishing the MISEV guidelines; The editorial process
Source: J Extracell Vesicles. 2024 Feb 15;13(2):e12415. doi: 10.1002/jev2.12415 (PMC10867691; doi:10.1002/jev2.12415)
Supplement: Supplementary file 2 — Supplementary Information [file JEV2-13-e12415-s001.docx]

| **Full anonymised revision 1 comments for MISEV2023** | |
| --- | --- |
| 1 | Overall an excellent revision of this important document.  Two small observations:   1. Section 5.1 page 22 top line. Limit of Detection (LOD) is discussed here in the text and the importance of reporting. Though it is acknowledged that some techniques would be difficult to report LOD. Therefore I would suggest altering the first recommendation bullet point to ‘report LOD where known’ or similar text. 2. Table 2 – an excellent addition – could it be checked for common colour blindness issues, especially the yellow-peach-red sections might be very similar for some types of colour blindness. Might require asking Wiley to use certain tones. Or adding text somewhere in the table in each section ‘green’, ‘yellow’, ‘amber’, ‘red’. |
| 2 | Line 750: add “enrichment” in addition to separation/concentration  Line 754: delete “now”  Line 761: delete “strict”  The first sentence of 4.1 can be omitted  Are all abbreviations introduced (for example CCM, Eps, and more)?  Line 769: consider including “enrichment”, actually this goed for the entire section…  Line 774: the statement on commercial kits requires a reference  Line 778: “including 3, 10, 100, and 1000 kDa” may be omitted  Line 801: include cut off (in kDa)  4.2: “perfect separations are not achieved”….. this is common for all/most techniques, including TFF  Line 823: “but…..by dUC” can be omitted (double)  Line 830-837: I don’t see the sense for including this  In section 4.3: maybe include that, depending on the purity, a density-range rather than an exact density ca be expected  In section 4.4, illustration of optimal, or commonly used materials/pore sizes could be helpful  Line 972: mention tetraspanins  In 4.6, it has to be made very clear that (specific) sub-populations are isolated by this approach  4.7: in line 1021, delete “and caution”  Line 1022-1038: maybe move down a bit, or even consider deleting  Line 1049-1063: these strong statements require references, there is not a single one in this bit  Section 5  Line 1080: include contamination, for example by detached or broken cells  In the quantification section: the use of dilution series is essential!!! For reporting: mention dilution series (yes/no), mention reference material (beads/liposomes)  Section 5.2: the title is a bit strange. Quantification? Maybe consider determining/analysis?  Section 5.7: consider mentioning the multiplex flow cytometry kits for EV characterization (see also [Systematic Methodological Evaluation of a Multiplex Bead-Based Flow Cytometry Assay for Detection of Extracellular Vesicle Surface Signatures - PubMed (nih.gov)](https://pubmed.ncbi.nlm.nih.gov/29951064/))    Section 5 is in general very good, this is maybe even the core of MISEV |
| 3 | [1] I would suggest to add the following words in the section of 4.1 EV concentration.   - (ultra)centrifugation (Line 757 / 760, Page 14) - Molecular weight cut-off (Line 762, Page 14) - Reporting recommendations: for concentration, report the following: (Ling 763, Page 14) Molecular weight cut-off size, Temperature during concentration   [2] I would suggest to add the following words in the section of 4.6 Charge and molecular recognition-based separations (Line 972, Page 19)   - Elution process (elution buffer composition, time)   [3] I would suggest to add the following sentence in the section of 5.3 Quantification of total protein (Line 162, Page 23)   - Report whether intact or disrupted EVs are used   [4] I would suggest to add the following sentence in the section of 5.5 Quantification of total RNA (Line 193, Page 23)   - Report whether DNase is pre-treated |
| 4 | All sections are excellent,  Occasional issues (below) but they are minor comments aimed at getting it absolutely correct.    Chp4  **Line722** – I have a slight issue around this concept of “recovery” vs “specificity” (fig2) when comparing different methods of isolation.  Because the recovery measurement relies on knowing the EV amount before vs after the isolation method. In most cases this “before” measurement is not strictly possible (not ev specific)- especially with respect to complex biofluids.    I advise slightly more guarded wording such as “estimated recovery” and perhaps an explanation of this choice terminology.    For fig 2, I think (a), (b), (c) labelling on the fig and corresponding legend would be useful.    **Line 739**- precipitants, do they really “eliminate water molecules” ?    **Line753**- “without clogging of the filter”;  TFF does suffer from formation of a gel layer on the filter surface, it is not a route to eliminate this problem, it **reduces** the clogging of filter pores vs traditional filter systems.    **Line-756**.  The lamparski paper (doi: 10.1016/s0022-1759(02)00330-7.) is the earliest TFF EV-for clinical application ref, not the one given    **Line835**- “It is good practice to weigh given volumes of each fraction to confirm final density”,   but measures of refractive index which are proportional to density are also useful.    **Line837**- “Recovery after density gradient and fraction washing is relatively low”.    I refer to earlier comments- the absolute amount recovered is low, but the recovery (efficiency of isolation) is unknowable. Might also therefore state that density-UC is more suited to an analytical scale of isolating EVs as opposed to preparative larger scale processes.    **Line896**- ‘FFE combines flow with electrophoresis, adding separation by, e.g., isoelectric point (Preußer et al. 2022). Introducing separation buffers with different pH or other characteristics across the separation channel allows high-resolution separation of different EV and other EP populations. FFE can be done at various scales.’   - Wrt gentle-ness , do these pH gradients etc have an impact on the vesicle- should this be taken account of.   Sorry don’t know much on these approaches but the pH thing suggests its unlikely to be fully inert as a process.    Addition here would be consistent with later line946 discussing elution from affinity bound evs     **Line907** recommendations-  I believe these systems have detector systems (UV abs etc) ? which should also be reported surely.  **Chp5- this is really great section-**  **Line1166**-   discussing recommend reporting- that protein measures should be on the linear part of the curve- I suggest this should also be true for particle measurements surely !    **Line1191** ish- this section discusses RNAse DNAse treatment. -assuming these are contaminants of ev preps.   Is there a danger here of removing nucleic acid associated with the ev (ie corona) and dismissing these elements as contaminants >    Really pleased with how this is looking overall.  Congrats to all on this collective effort. |
| 5 | Some very minor comments from me on the sections below:     - Lines 768-772 needs some references included to back up some of the comments – eg for the last sentence - Line 807 – I don’t think the term ‘perfect separations’ is a good term to use and this sentence is not referring to the separation of specific particles – eg ‘perfect separation’ of what? - Line 909 – SEC doesn’t need to be in bold (as it is in the line above) - Line 926 – need a reference for free-flow electrophoresis - Line 937 – ‘exquisitely’ is not needed - Line 1243 – I would not call the 2013 ISEV RNA Position paper the most recent one! Remove ‘most recent’ |
| 6 | The authors have adjusted according to my previous comments on Chapter 5 – EV characterization. And for the new chapter, Chapter 4 – EV separation and concentration I have no further comments I think the text is educational and well balanced. Great job by the authors. |
| 7 | General comment:   - The readability of the manuscript and the balance between different topics has largely improved   I have been asked to focus my comments on sections 4 and 5.  Section 4:   - No further comments   Section 5:   - 5.2, lines 177-179: in my opinion this statement is too generalized. Especially the size of EVs is very much dependent on species, cell types, body fluids, isolation method, etc - 5.9, lines 307-309: Contains too little concrete info relative to the other topics discussed   In addition I have a few comments on Section 3:   - 3.9, lines 646-647: ‘milk components that share EV characteristics': here ‘cells’ is mentioned as an example, but cells don’t overlap with EVs in size and/or density. I think it is better to state that besides components that overlap in size/density with EVs, also components that may lead to artificially generated small particles overlapping with EVs during storage/processing need to be removed - 3.12: very important to explain that freezing PBS without addition of co-solutes like trehalose leads to severe pH drops (this may severely damage EV integrity). This is not widely known in EV community and worth explaining. There are several papers that can be referred to here, e.g. <https://pubmed.ncbi.nlm.nih.gov/31087169/> |
| 8 | **Comments to Chapter 4, “EV separation and concentration”**  Very well written and I have no critical comments on this chapter.  Minor points,  Page 13, line729: directly in a biological fluid (Duijvesz et al. 2015; Woud et al. 2022).  Corrected to : directly in a biological fluid ( Logozzi et al., 2009; Yoshioka et al., 2014; Duijvesz et al. 2015; Woud et al. 2022).  Refs:  Logozzi et al., 2009 as  Logozzi M, De Milito A, Lugini L, Borghi M, Calabrò L, Spada M, Perdicchio M, Marino M L, Federici C, Iessi E, Brambilla D, Venturi G, Lozupone F, Santinami M, Huber V, Maio M, Rivoltini L, Fais S. High levels of exosomes expressing CD63 and caveolin-1 in plasma of melanoma patients. 2009. PloS one, 4 (4), e5219-5219.  Yoshioka et al., 2014 as  Yoshioka Y, Kosaka N, Konishi Y, Ohta H, Okamoto H, Sonoda H, Nonaka R, Yamamoto H, Ishii H, Mori M, Furuta K, Nakajima T, Hayashi H, Sugisaki H, Higashimoto H, Kato T, Takeshita F, Ochiya T. 2014. Ultra-sensitive liquid biopsy of circulating extracellular vesicles using ExoScreen. Nat Commun, 5:3591  Page 14, Line 756:  large-scale EV production, e.g., for therapeutic applications (Busatto et al. 2018). Finally, concentration can also be;  added the explanation of a new methods as follows:  large-scale EV production, e.g., for therapeutic applications (Busatto et al. 2018). The ultrafast-isolation system (EXODUS) to purify EVs automatically by undergoing negative pressure oscillation and membrane vibration driven by double coupled harmonic oscillator (Che et al., 2021) is applicable for large scale purification. Finally, concentration can also be  Ref: Chen et al., 2021 as  Chen Y, Zhu Q, Cheng L, Wang Y, Li M, Yang Q, Hu L, Lou D, Li J, Dong X, Lee LP, Liu F. Exosome detection via the ultrafast-isolation system: EXODUS. 2021. Nat Methods,18:212–218.  **Comments to Chapter 5, “EV characterization”**  Very well written and I have no critical comments on this chapter. |
| 9 | I have now reviewed the sections requested and find that it is well written and almost all aspects and open questions are discussed.  In my opinion, the manuscript can be published in current form without delay, |
| 10 | Overall, I do feel that this version of the MISEV guidelines meets the expectations and provides adequate guidance for newcomers and by extension all EV researchers. Below a summary of my thoughts and some specific minor comments.    **Main comment 1 related to Section “EV separation and concentration”: general lack of guidance on EV separation and concentration.**  The EV separation and concentration section has been very much improved. Manuscript is now overall adequately describing the general concepts and considerations of both the concentration/separation and characterization steps that are typically involved in EV analysis. So I feel that newcomers in the field are adequately informed and receive adequate guidance to other dedicated manuscripts such as previous versions of the MISEV guidelines or position papers etc. Also all additional minor comments raised have been properly integrated.  Indicated summaries and reporting recommendations are an added value and overall clear in the EV “separation and concentration” section. One may consider a similar format for the EV “characterization section” which tends to intermingle summaries and reporting recommendations.  **Main comment 2 related to Section “Collection and pre-processing”: lack of coherence between the different subsections.**  This section is now adequately balanced between the different subsections. Also all additional minor comments raised have been properly integrated.  **Main comment 3 related to Section “Characterization”: lack of logic in the ordering of the methods.**  This section is now more logically arranged (rather than alphabetically). Also all additional minor comments raised have been properly integrated.  **Specific comments:**  -Line 424: Dhondt et al., 2020 concerns urine and should be removed in this particular section dealing with blood.  -Line 847: not ‘in the middle of the channel’ but ‘towards the middle of the channel’. Particles usually do not reach the middle of the channel but stay within the 10% of the channel close to the membrane.  -Line 860: ‘AF4 is also not a preparative technique.’ What actually is meant here? Also by AF4, fractions can be collected, concentrated and processed. |

Supplementary Table 1. Full anonymised revision 1 comments for MISEV2023.
